# Complications from Stereotactic Body Radiotherapy for Lung Cancer

**DOI:** 10.3390/cancers7020820

**Published:** 2015-06-15

**Authors:** Kylie H. Kang, Christian C. Okoye, Ravi B. Patel, Shankar Siva, Tithi Biswas, Rodney J. Ellis, Min Yao, Mitchell Machtay, Simon S. Lo

**Affiliations:** 1 School of Medicine, Case Western Reserve University, Cleveland, OH 44106, USA; E-Mail: Kylie.Kang@case.edu; 2 Department of Radiation Oncology, University Hospitals Seidman Cancer Center, Case Comprehensive Cancer Center, Case Western Reserve University, Cleveland, OH 44106, USA; E-Mails: Christian.Okoye@uhhospitals.org (C.C.O.); Ravi.Patel@uhhospitals.org (R.B.P.); Tithi.Biswas@UHhospitals.org (T.B.); Rodney.Ellis@UHhospitals.org (R.J.E.); Min.Yao@UHhospitals.org (M.Y.); Mitchell.Machtay@UHhospitals.org (M.M.); 3 Division of Radiation Oncology and Cancer Imaging, Peter MacCallum Cancer Centre, East Melbourne, Victoria 3002, Australia; E-Mail: Shankar.Siva@petermac.org

**Keywords:** stereotactic body radiation therapy (SBRT), stereotactic ablative radiotherapy (SABR), non-small cell lung cancer (NSCLC), toxicity, complications

## Abstract

Stereotactic body radiotherapy (SBRT) has become a standard treatment option for early stage, node negative non-small cell lung cancer (NSCLC) in patients who are either medically inoperable or refuse surgical resection. SBRT has high local control rates and a favorable toxicity profile relative to other surgical and non-surgical approaches. Given the excellent tumor control rates and increasing utilization of SBRT, recent efforts have focused on limiting toxicity while expanding treatment to increasingly complex patients. We review toxicities from SBRT for lung cancer, including central airway, esophageal, vascular (e.g., aorta), lung parenchyma (e.g., radiation pneumonitis), and chest wall toxicities, as well as radiation-induced neuropathies (e.g., brachial plexus, vagus nerve and recurrent laryngeal nerve). We summarize patient-related, tumor-related, dosimetric characteristics of these toxicities, review published dose constraints, and propose strategies to reduce such complications.

## 1. Introduction

Few recent developments in the treatment of non-small cell lung cancer (NSCLC) have matched the impact that stereotactic body radiotherapy (SBRT), also referred to as stereotactic ablative radiotherapy (SABR), has had on outcomes in early stage, medically inoperable NSCLC [1]. Prior to the advent of SBRT, these NSCLC patients had dismal outcomes, with poor local control rates, and 5-year survival rates of 20%–30% with conventionally fractionated external beam radiation (EBRT) [2,3,4]. Although escalation of doses above 80 Gy improved local control and survival, dose limiting toxicities were reached at doses of 84–90 Gy [5,6,7]. Intensification of treatment through the addition of concurrent chemotherapy and hypofractionation were also attempted with limited success; however, improvements in localization and radiation delivery allowed for hypofractionated, dose escalated regimens to be delivered safely [8], with 2-year local control rates above 90% [9,10].

Currently, SBRT is the treatment of choice for early stage, node negative NSCLC in patients who are either medically inoperable or refuse surgical resection. These stereotactic techniques reduce target uncertainty and planning target volume (PTV) margins while improving dose gradients across tissue, allowing delivery of high cumulative doses and improving local control while reducing dose related toxicity by minimizing exposure to organs at risk (OAR) [11]. While retrospective comparisons of patients treated by surgery and SBRT are limited by an imbalance of poor prognostic factors in SBRT cohorts [12], after statistical adjustments for these differences, similar outcomes between SBRT and both limited sublobar resection and lobectomy have been reported [12,13].

While treatment of early stage NSCLC with SBRT has proven to be an effective treatment modality with acceptable toxicity, careful patient evaluation, simulation, treatment planning, and eventual radiation delivery is required to minimize complications. With this in mind, we review the literature on treatment considerations for delivering lung SBRT.

## 2. Centrally Located Tumors

**Figure 1:**
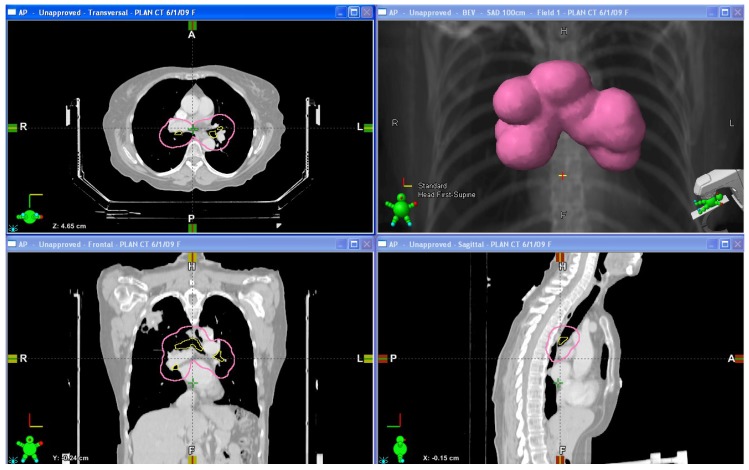
Diagrams of the proximal bronchial tree and the surrounding 2 cm avoidance zone. Upper left: Axial view; Lower left: Coronal view; Upper right: 3-dimensional representation of the avoidance zone; Lower right: Sagittal view.

For early stage NSCLC, centrally located lesions are defined as any lesion existing within a 2 cm zone surrounding the proximal bronchial tree, from the carina to the lobar and lingular bronchi [14] (Figure 1). Previously, central lesions treated with traditional SBRT doses have been shown to cause excessive toxicity, as was shown in a Phase II study from Indiana University where patients with centrally located (hilar/pericentral) tumors treated to 60 to 66 Gy in three fractions had an 11-fold higher risk of developing grade 3–5 toxicities when compared to similarly treated peripherally located tumors [10]. Updated results from this study [15] at a median follow up of 50.2 months confirmed a high incidence of grade 3 to 5 toxicity (27.3%), almost three times the rate for peripheral lesions (10.4%). Other studies have had similar observations. For instance, a phase I single fraction dose escalation trial for patients with primary or metastatic lung lesions by Le *et al.* reported late treatment-related toxicities in 25% of patients, of which the majority of overall toxicity (62.5%) and all three grade 5 toxicities (pneumonitis/pleural effusion, tracheoesophageal fistula, and pulmonary embolism/recall pneumonitis) were seen in centrally located tumors [16,17]. Thus, centrally located tumors represent a high-risk tumor location, predisposing such patients to a unique toxicity profile.

## 3. Central Airway Toxicities

Given the proximity of centrally located lesions to major airways, patients with these tumors are at higher risk for a dose-related major airway toxicity and consequent atelectasis, stenosis/stricture, airway necrosis, and/or fistula formation. Although its pathogenesis is poorly understood, airway toxicity has been hypothesized to be caused by a dose-dependent radiation-induced damage of the bronchial wall, leading to fibrosis and consequent stenosis and stricture. Data from more traditional conventionally fractionated radiation have showed narrowing caliber of mainstem bronchi, as measured by post-treatment CT as early as three months after high dose EBRT to doses of ≥73.6 Gy [18]. The data for central airway toxicity for SBRT is more limited; however, a retrospective study by Song *et al.*, showed complete or partial bronchial stricture in 8/9 (89%) patients with central tumors treated with SBRT (at doses of 40–60 Gy in 3–4 fractions) at a median follow-up time of 26.5 months [16]. Perhaps data from high dose rate (HDR) brachytherapy can provide the greatest information about central airway toxicity from ablative radiation doses. A study by Speiser *et al.* examined radiation reactions for a total of 342 patients treated with endobronchial brachytherapy at doses of 750–1000 cGy at 5–10 mm depth for three fractions [19]. For the entire cohort, there was a 12% bronchial stenosis rate with higher rates seen in patients with large cell histology, treated with curative intent, prior laser photoresection, and concurrent EBRT [19].

More severe central airway late toxicity could lead to complete bronchial stenosis with resultant atelectasis, bronchial necrosis, or fatal hemoptysis. A retrospective study by Karlsson *et al.* demonstrated findings of radiation-induced atelectasis in 24.3% of patients with prescribed doses of 20–50 Gy in 2–5 fractions for tumors close to the bronchial tree [20]. On subsequent survival hazard function analysis, the median dose to 0.1 cc of bronchial tree in patients who developed atelectasis was 210 Gy_3_ *vs.* 105 Gy_3_ in 2 Gy equivalent doses for patients who did not develop atelectasis (*p =* 0.031). Another study by Rowe *et al.* described one bronchial necrosis-related hemorrhage and death occurring 10.5 months after SBRT to a 5.7 cm metastasis abutting the left mainstem bronchus, with the area of necrosis having received a maximum dose above the dose prescribed [21]. Corradetti *et al.* presented a similar case of central-airway necrosis and subsequent fatal hemoptysis in a NSCLC patient receiving a fractionation scheme of 50 Gy in five fractions [22].

## 4. Esophageal Toxicity

Esophageal toxicity is another well-known complication of radiotherapy for centrally located tumors treated with SBRT [23], ranging from mild esophagitis to stricture, perforation, and/or tracheoesophageal fistula [9,24]. Previous data from a number of detailed analyses of conventional EBRT suggest that the volume of esophagus exposed to higher doses of radiation (Vd) is the most meaningful metric. For instance, Palma *et al.* found that on multivariable analysis, V60 formed the best predictive model for radiation esophagitis following 3D-CRT or IMRT (for ≥grade 2, OR 1.34 per 10% increase, *p <* 0.001; for ≥grade 3, OR 1.33 per 10% increase, *p <* 0.001) [25]. Interestingly, even when staying at or below previously reported safe thresholds (limiting the esophagus in a single fraction to D_5cc_ of 14.5 Gy, D_2cc_ of 15 Gy, and to D_max_ of 19 Gy) [26,27], SBRT fractionation regimens could still have single-fraction biologically effective doses (SFBEDs) to the gross tumor volume (GTV) exceeding these thresholds (calculated via linear quadratic (LQ) modeling), resulting in esophageal adverse events [28]. For instance, the two occurrences of high-grade esophageal toxicity events (tracheoesophageal fistula and esophageal perforation) in the study by Abelson *et al.* occurred at dose-volume points within or near the safe thresholds, at SFBED to D_5cc_ of 16.5 and 11.4 Gy, to D_2cc_ of 18.2 and 14.1 Gy, and to D_max_ of 21.0 and 18.5 Gy, respectively, by LQ modeling [28].

Additionally, post-SBRT administration of chemotherapy can be an important modifier of radiation sensitivity. One patient in a study by Abelson *et al.* who experienced grade 5 toxicity received adjuvant chemotherapy three months after SBRT (25 Gy in one fraction), consisting initially of carboplatin and paclitaxel and then switched to gemcitabine two cycles later, at which time the patient developed (within the high-dose radiation volume) a tracheoesophageal fistula and ultimately fatal hemoptysis [28]. It is possible that the post-SBRT chemotherapy contributed to the patient’s adverse events, especially given that chemotherapy in the setting of conventionally fractionated radiotherapy has been found to increase the risk of esophageal toxicity [29]. Although the mechanism of fistula formation is poorly understood, it is likely related to the impaired angiogenesis leading to delayed and dysfunctional wound healing [30], as well as predisposition to fistula formation by radiation and chronic inflammation [31]. There is a new clinical trial (NCT02319889) looking at Abraxane (nab-paclitaxel) after SBRT which should better elucidate the potential role of SBRT and combination chemotherapy in improving treatment response as well as toxicity [32].

## 5. Vascular Injury

SBRT to central tumors also increases the dose to the central vascular structures, particularly the aorta. Although typically thought of as relatively radio-resistant, elevated doses seen in SBRT and the re-irradiation setting may lead to severe toxicity. In a landmark study by Evans *et al.*, patients were evaluated for dosimetric correlates for aortic toxicities, defined as hemoptysis secondary to aortic damage, exsanguination secondary to aortic rupture, aortic aneurysm in the irradiated region, or aortic dissection [33] (Table 1). At a median follow up of 42 months, 2/35 (5.7%) patients had developed grade 5 aortic toxicity, ranging up to 39 months after the last radiation treatment [33]. In their dosimetric analysis, there was a 25% rate of grade 5 aortic toxicity for patients receiving maximum composite doses to 1 cm^3^ of the aorta ≥120 Gy *versus* 0% rate for those receiving <120 Gy (*p =* 0.047) [33].

**Table 1:** Dosimetric considerations.

Endpoint	Organ	Dosimetric Constraint (Comment)	Study
Vocal Cord Paralysis	Recurrent Laryngeal Nerve (RLN), Vagus Nerve (VN)	Of 12 patients with significant dose to either the RLN or VN, 2 patients developed vocal cord paresis, at a cumulative single fraction equivalent dose (SFED_3_; α/β = 3 Gy) to VN of 64.5 Gy and 16 Gy and SFED_3_ to the RLN 15.3 Gy and 19.5 Gy.	Shultz *et al.*, 2014 [34]
Aortic Toxicity	Aorta	Recommended dose threshold of 120.0 Gy as a raw dose, 90.0 Gy when dose is corrected for long-term recovery during retreatment interval	Evans *et al.*, 2013 [33]
Brachial Plexopathy	Brachial Plexus	Doses >26 Gy in 3–4 fractions resulted in an increased 2 year risk of brachial plexopathy, with similar cutoffs noted for BED >100 Gy_3_ and SFED-4 >15 Gy	Forquer *et al.*, 2009 [35]

Other potentially life threatening vascular complications from SBRT include hemoptysis and pulmonary hemorrhage. Potential risk factors for its occurrence include central location of tumor (OR = 3.003, *p =* 0.113) [36] (OR = 6.976, *p =* 0.017) [37], squamous histology (OR = 5.491, *p =* 0.040) [36], baseline major tumor cavitation (OR = 17.878, *p =* 0.001) [36], and suspicion of endobronchial involvement (OR = 12.8, *p =* 0.024) [38].

Of these risk factors, central location of tumor is a notable cause of fatal hemoptysis. Death following treatment of centrally located tumors, even at lower dose per fraction, has been reported in many studies and is related to inclusion of the esophagus and/or bronchus in the high dose volume, which leads to bronchial stricture or tissue ulceration and necrosis [39]. For example, Milano *et al.* cite a case of hemoptysis following SBRT for a central NSCLC that exposed the bronchus to a cumulative dose of 98 Gy [40]. Moreover, retreatment of central lung lesions with SBRT also increases the risk for hemoptysis. Oshiro *et al.* reported a patient who previously received two courses of thoracic radiotherapy (brachytherapy and SBRT) with grade 5 (fatal) hemoptysis 18 months post-SBRT with BED_10_ of 87.5 Gy [41].

## 6. Spontaneous Pneumothorax and Other Pulmonary Toxicities

Another rare complication of SBRT for NSCLC is spontaneous pneumothorax, which may occur as a result of radiation-induced pulmonary changes, apical pleural injury, and/or parenchymal injury [42]. Although previously seen predominantly in patients treated for Hodgkin’s lymphoma for whom large volumes of pleura were within the radiotherapy (RT) field, Onishi *et al.* described a case of asymptomatic spontaneous pneumothorax detected during a routine follow-up by chest X-ray and chest computed tomography (CT) two months after receiving SBRT for NSCLC to separate right upper and right lower lobe lesions [42]. Postulated contributing factors include tumor related air trapping and consequent alveolar space rupture, and RT-induced dense pulmonary/pleural fibrosis leading to rupture of subpleural blebs [43]. However, given the rarity of such a complication, specific data on its incidence and/or risk factors for its development are lacking.

Although concern exists for changes in post treatment pulmonary function (PF) after SBRT, data suggests these changes may only be only of minimal clinical relevance. Investigators from the Indiana studied the effects of SBRT on PF, and found no significant long-term change in FEV_1_, FEV_1_%, or DL_CO_ after treatment [44,45]. Similar studies [9,46,47] also suggest minimal PF changes following SBRT, comparable to those expected from physiologic aging [9,44,46,47,48]. Furthermore, there has been no determination of a minimum PF necessary for the safe practice of SBRT [46]. Thus, baseline PF alone may not be an absolute contraindication to treatment with SBRT [49], and dose reduction aimed at preserving long term PF may be unjustified [44].

## 7. Radiation Pneumonitis

Radiation pneumonitis (RP) is one of the major toxicities which limits the maximal radiation dose that can be safely delivered to thoracic tumors [50], with severity ranging from asymptomatic cases detected only radiographically to clinically evident cases involving cough, shortness of breath, and fever. In severe cases, there may be dense fibrotic lung changes and respiratory compromise requiring supplemental oxygen or assisted ventilation [51]. Reported rates of post-SBRT RP requiring clinical intervention range from 0% to 29% [52,53,54,55], with the occurrence of severe (≥grade 3) RP usually uncommon, even among patients with compromised lung function [56,57]. This is likely due to the small target sizes and limited planning margins allowed by SBRT, thus minimizing the volume of normal lung tissue exposed to escalated doses [56,57]. Nevertheless, life-threatening toxicities have been reported in up to 12% of cases in various studies [10,53]. Therefore, understanding the risk factors for its occurrence, including dosimetric, diagnostic (e.g., biological, radiological), and patient-related (e.g., pulmonary-related comorbidities) predictors, are warranted.

### 7.1. Dosimetric Predictors of RP

The best supported dosimetric correlates of RP is mean lung dose (MLD), followed by V5 and V20 (percentage of the lung volume receiving >5 Gy and >20 Gy, respectively) [8,56,58,59]. Barriger *et al.* noted unadjusted total lung mean doses above 4 Gy and V20 greater than 4% being associated with increased RP on univariate analysis [8]. Interestingly, Ong *et al.* showed the best predictor of RP to be the contralateral lung V5, with all patients in their series developing RP with a contralateral lung V5 >26% [59].

Additional predictors of RP relate to increasing radiation target volume, as larger tumor sizes result in larger volumes of lung treated to higher doses [59]. Similarly, the risk of RP is also related to the conformity index (CI), defined as a ratio of the volume encompassed by the prescription isodose line (IDL) and volume of the PTV [51,58]. Although only indirectly related to the treatment volume, applying constraints to this additional parameter ensures the high dose region is restricted to the area immediately adjacent to the target. Clinically, Yamashita *et al.* found that none of the traditional dosimetric variables, including V5, V10, V20, and MLD, correlated with the CI, and that high CI values correlated significantly with RP occurrence (*p =* 0.0394) [53].

The anatomical location of the treatment region within the lung may also correlate with the development of RP. Kayas *et al.* reported that the portion of the lung below its geometric mid-line appears to be more radiosensitive than the upper portion and consequently more susceptible to RP [60]. This finding is consistent with those of other studies, where inferior tumor location [50,61] and ipsilateral lower lobe mean effective dose [62] correlated with RP. This phenomenon is likely explained by the greater target cell density [63] and greater functional importance of the lower lung [64], resulting in more severe symptoms upon damage. One must also take into account the greater respiratory motion seen for lower lobe tumors, which may expose larger volumes to radiation dose during treatment delivery, particularly in the absence of robust motion management [62].

### 7.2. Diagnostic Predictors of RP

Along with dose-volume factors, several biomarkers and radiologic imaging findings can help predict the risk for severe RP after SBRT [65]. Higher levels of the biomarkers serum Krebs von den Lungen-6 (KL-6), a circulating mucin-like glycoprotein produced and secreted from type II pneumocytes, and surfactant protein-D (SP-D) were associated with increased risk of severe RP after receiving SBRT [66,67]. Mechanistically, serum levels of KL-6 are dependent on the number of regenerating pneumocytes and integrity of the alveolar-capillary membrane [68], which may be compromised before treatment and/or in the setting of RP. In addition to elevated pre-treatment KL-6 levels (>500.0 U/mL), significant independent factors of high grade RP noted in the Yamashita experience included elevated levels of SP-D (>110.0 ng/mL) and presence of an interstitial pneumonitis (IP) shadow on pre-treatment CT.

These markers have also shown correlations with RP when measured after initiation of RT. Patients with elevated post treatment KL-6 levels in the months after RT relative to baseline (>1.5 to 1.7 times the pretreatment value) have shown correlations with symptomatic, grade 2 to 3 RP, values of which decrease in response to steroid use [67,69]. In a separate cohort, radiographic changes were seen in all patients who developed grade 3 RP after SBRT, the incidence of which was 75%, 40%, and 1.2% in patients with imaging findings at one, two, and greater than three months after SBRT, respectively [70]. Although these markers await prospective validation and widespread clinical adoption, they demonstrate the potential for individualized patient-specific markers to predict clinical toxicities in thoracic SBRT.

### 7.3. Patient-Related Predictors

Many patient-related factors have been investigated for their relationship with the incidence of RP, including co-morbid pulmonary disease, smoking history, and female gender. Underlying subclinical interstitial lung disease (ILD), defined by characteristic chest CT findings, may predispose patients to uncharacteristically extensive and fatal RP extending beyond the irradiated field [71]. In contrast, a retrospective series of patients with GOLD stage III-IV Chronic Obstructive Pulmonary Disease (COPD) treated with SBRT demonstrated low rates (1.7%) of grade 3 RP with risk adapted fractionation schemes [72]. The presence of pulmonary emphysema also was not found to predict for RP after SBRT, with at least one group demonstrating lower rates compared to those with normal function [71,73]. Although these data support the safety and efficacy of SBRT in this patient population, the underlying comorbid disease appears to dictate overall survival, as patients with GOLD IV COPD had worse median survival than those with GOLD III COPD (17 *vs.* 36 months, log-rank *p =* 0.01) [72].

Smoking also appears to be inversely correlated with the development of RP, particularly with regards to smoking status at the time of RT [74] and pack-years smoked [75]. Possible explanations for the protective effect of smoking on the incidence and degree of RP include possible reduction in radiation-induced inflammation [76] and increases in smoking-associated pulmonary and/or plasma glutathione, and consequent prevention of oxidant lung injury [77].

At least one study did show that female gender was a significant factor for predicting grade 2 RP in multivariate analysis [78], with a potential confounding of reduced total lung volume among females, which may result in relatively higher doses delivered to the remaining normal lung [78].

## 8. Chest Wall and Skin Toxicities

Chest wall toxicities are usually associated with SBRT for peripherally located lung tumors [79,80], and include skin toxicity, rib fracture, and chronic chest wall pain [57]. Given the dosimetric nature of chest wall toxicities, patients with tumors more than 1–2 cm from the chest wall and 5 cm from the posterior skin are at very low risk of toxicity [81].

### 8.1. Chest Wall Pain and Rib Fracture

Chest wall pain (CWP) after SBRT, especially for treatment to lesions in close proximity to the chest wall, has been reported in 5%–25% of patients [80,81,82,83] and can present with or without demonstrable rib fracture. Although the mechanism of chest wall pain following SBRT is poorly understood, possible target tissues responsible for this toxicity include the underlying large peripheral intercostal nerves [84] and bone [85]. Patient-related factors may also increase the risk of CWP, including younger patient age [86] and continued smoking [81]. Additional reports demonstrate obesity as a significant risk factor for CWP [82], with one study showing a nearly double increased in risk of chronic CWP for patients with ≥29 BMI compared to patients with <29 BMI (27% *vs.* 13%, respectively; *p =* 0.01) [82]. Among the group with elevated BMI, diabetes mellitus (DM) was also highly correlated with pain [82].

It appears that CWP may be at least partially independent of direct rib injury, as Andolino *et al.* reported only 19% of CWP episodes coincided with a documented rib fracture [80], and most patients in a series by Creach *et al.* who developed rib fracture did not report CWP prior to the rib fracture [87]. Additionally, the characteristic timing of onset after RT appears to differ between the two toxicities (Table 2). In terms of rib fracture alone, there is a strong association between individual rib dose-volume histogram parameters and fracture risk in the small-volume/high dose region, such as V60 [88,89]. Other reported parameters for chest wall toxicity and radiation-induced rib fracture include chest wall dose of 30 Gy being received by >30 to 70 cm^3^ [80,81,82,84,89], dose to 2 cm^3^ of the chest wall [84,88], and maximum dose to the rib or chest wall >50 Gy [80]. Limiting chest wall toxicities may also be possible through more protracted hyperfractionated regimens, as some investigators have found comparable local control with reduced CWP incidence (<6%) [90].

**Table 2:** Chest wall and related toxicity characteristics.

Toxicity	Incidence	Timing	Dosimetric Correlates			Study
			Volume	Dose	Fraction	
Chest Wall Pain	30% risk (Range: 10% to 44%)	12.6 months post-SBRT (Range: 4.3–35.9 months)	30 cc of chest wall	30 Gy	3 (Range:3–5)	Dunlap *et al.*, 2010 [89] Mutter *et al.*, 2012 [84] Creach *et al.*, 2012 [87]
Rib Fracture	5% risk	19.2 months post-SBRT	2 cc of rib	27 Gy	3	Pettersson *et al.*, 2009 [88]
	50% risk		2 cc of rib	50 Gy	3	Creach *et al.*, 2012 [87]
Skin Toxicity	1.2%–14%	3–6 weeks post-SBRT	<10 cc volume, volume maximum of 30 Gy	6 Gy/fraction	5	RTOG 0813 [91] Hoppe *et al.*, 2008 [92]
			maximum point dose of 32 Gy, maximum posterior skin dose ≥50% of actual prescribed dose	6.4 Gy/fraction	5	

### 8.2. Skin Toxicity

Although observed in a limited number of patients, selective subsets remain at elevated risk for skin toxicity, including those with a large body habitus [57], and posterior tumors location with limited distance between the lesion and overlying skin [82,92]. Additional dosimetric risk factors include treatment delivery through a limited number of coplanar beams, maximum skin doses 50% or greater than the prescribed dose [92], and among lesions within 2.5 cm of the chest wall, volume of the chest wall receiving 30 Gy (V30) (<50 mL, 22% *vs.* ≥51 mL, 44%, *p =* 0.02) [82].

## 9. Brachial Plexopathy

Tumors located in the apex of the lung pose unique local treatment challenges due to their close proximity to the brachial plexus and resulting radiation-induced brachial plexopathy (RIBP), with symptoms of upper extremity paresthesias, motor weakness, and neuropathic pain [93]. Mechanistically, RIBP is thought to be due to demyelination leading to axon loss [94], consistent with the observation that multiple traumas, including that from tumor invasion and/or previous surgery, may reduce the threshold for development of symptoms [94,95,96].

Limited dosimetric studies for RIBP indicate a threshold effect. Forquer *et al.* found that SBRT using absolute brachial plexus doses >26 Gy in three to four fractions resulted in an increased 2-year risk of brachial plexopathy compared to doses ≤26 Gy (46% *vs.* 8%, *p =* 0.04), with similar cutoffs noted for BED >100 Gy_3_ (*p =* 0.04) and SFED-4 (single fraction equivalent dose, where D_q_ = 4 Gy) >15 Gy (*p =* 0.06) [35]. Chang *et al.* similarly found that centrally located lesions treated to doses of 50 Gy in four fractions had a risk of RIBP limited to those where brachial plexus D_max_ >35 Gy and V30 >0.2 cm^3^ (*p =* 0.001) [97]. Given the limited experience with RIBP, practitioners should exercise caution when treating apical tumors.

## 10. Vagus Nerve Injury

Although rare, thoracic SBRT delivered to central structures may result in vagus nerve (VN) injury due to its location within mediastinum [34]. Anatomically, the left vagus nerve is at risk when left upper lobe lesions are treated given its close association with the lung parenchyma, while in the right hemithorax, the VN branches into the right recurrent laryngeal nerve (RLN), where it is at risk for injury as it drapes over the apical pleura and courses back toward the tracheoesophageal groove [34]. While data from Shultz *et al.* suggest that generally both VN and RecLN are relatively resistant to SBRT, 2 of 12 (17%) patients who received significant doses to either of these structures ultimately presented with observable nerve injury in the form of vocal cord paralysis from ipsilateral VN and RecLN injury (Table 1). Although no clear dose response was found, possible predisposing factors to toxicity include high cumulative doses due to re-irradiation in one patient, and pre-existing connective tissue disease (rheumatoid arthritis) in increased radiation sensitivity in another patient [34].

## 11. Ways to Avoid Complications

### 11.1. Patient Selection

The initial patient evaluation should include an evaluation of the patient’s functional status and any comorbid conditions that may impact the safety and efficacy of SBRT. Specifically, patients should be evaluated both functionally and radiographically for baseline pulmonary abnormalities, including IP shadows on pretreatment CT scans [66]. Although not used in routine practice, consideration may be given to evaluation of serum KL-6 and SP-D levels both before and after SBRT to identify those at higher risk for complications [67]. Although these markers await prospective validation, Yamashita *et al.* was able to show a reduction in pneumonitis risk (18.8% to 3.5% grade 4–5 RP) when patients with elevated levels of KL-6 and SP-D and obvious interstitial pneumonitis (IP) shadows on CT were excluded from receiving SBRT [66]. Additionally, practitioners should be aware of the timing of SBRT relative to any chemotherapy, where added caution may be necessary in the SBRT planning phase to avoid toxicity [28].

### 11.2. Simulation/Motion Management

In general, a very robust immobilization system [14] and an accurate pretreatment verification of PTV positioning and delivery of SBRT [57,97] are crucial in the prevention of inadvertent overdosing of OARs, though unnecessary immobilization may lead to added skin toxicity and thus should be avoided [92,98]. Respiration management can reduce the internal target volume (ITV) and consequently the normal tissue irradiated [65], while minimization of target uncertainty with techniques such as active breath control (ABC), cone-beam CT [99], and/or respiratory gating [100] can decrease PTV margins and thus toxicities associated with chest wall, mediastinal, and other soft tissue structures.

### 11.3. Treatment Planning

#### 11.3.1. Dose/Fractionation

Local control of lung tumors generally requires a biologically effective dose (BED) of at least 100 Gy [21,101,102,103,104]. Therefore, centrally located tumors require modulation of the dose size and/or fractionation to allow for safe delivery without increased toxicity [97]. Less intensive fractionation schemes for SBRT or risk-adapted approaches have been advocated regardless of tumor location within the chest [44,56,103,105,106,107,108]. Currently, 50 Gy delivered over five fractions has been widely adopted for the treatment of centrally located tumors [22,109], although more protracted and presumably safer regimens have been explored by several institutions [90,97,110]. As efforts to reduce toxicity may lead to unexpected changes in local control [22,24,101,103,111], cooperative group trials, such as Radiation Therapy Oncology Group (RTOG) 0813 [91], 0915 [112], and 0236 [113], have looked to build off of previous experiences by clearly defining the efficacy of SBRT regimens, while prospectively evaluating dose constraints and consequent toxicity with various fractionation schemes (Table 3).

**Table 3:** Published stereotactic body radiotherapy (SBRT) dose constraints.

Organ	Endpoint (≥Grade 3)	Dosimetric Constraints		Fractions	Prescription Dose (Gy)	Reference
		Volume	Constraint			
Esophagus	Stenosis/Fistula	<5 cc	11.9 Gy	1	34	RTOG 0915 [112]
		Max Point Dose	15.4 Gy			
		Max Point Dose	27 Gy	3	60	RTOG 0236 [113]
		<5 cc	18.8 Gy	4	48	RTOG 0915 [112]
		Max Point Dose	30 Gy			
		<5 cc	27.5 Gy	5	40–60	RTOG 0813 [91]
		Max Point Dose	105% of PTV Prescription			
Brachial Plexus	Neuropathy	<3 cc	14 Gy	1	34	RTOG 0915 [112]
		Max Point Dose	17.5 Gy			
		Max Point Dose	24 Gy	3	60	RTOG 0236 [113]
		<3 cc	23.6 Gy	4	48	RTOG 0915 [112]
		Max Point Dose	27.2 Gy			
		<3 cc	30 Gy	5	40–60	RTOG 0813 [91]
		Max Point Dose	32 Gy			
Great Vessels	Aneurysm	<10 cc	31 Gy	1	34	RTOG 0915 [112]
		Max Point Dose	37 Gy			
		<10 cc	43 Gy	4	48	RTOG 0915 [112]
		Max Point Dose	49 Gy			
		<10 cc	47 Gy	5	40–60	RTOG 0813 [91]
		Max Point Dose	105% of PTV Prescription			
Trachea and Large Bronchus	Stenosis/Fistula	<4 cc	10.5 Gy	1	34	RTOG 0915 [112]
		Max Point Dose	20.2 Gy			
		Max Point Dose	30 Gy	3	60	RTOG 0236 [113]
		<4 cc	15.6 Gy	4	48	RTOG 0915 [112]
		Max Point Dose	34.8 Gy			
		<4 cc	18 Gy	5	40–60	RTOG 0813 [91]
		Max Point Dose	105% of PTV Prescription			
Rib	Pain or Fracture	<1 cc	22 Gy	1	34	RTOG 0915 [112]
		Max Point Dose	30 Gy			
		<1 cc	32 Gy	4	48	RTOG 0915 [112]
		Max Point Dose	40 Gy			
Skin	Ulceration	<10 cc	23 Gy	1	34	RTOG 0915 [112]
		Max Point Dose	26 Gy			
		<10 cc	33.2 Gy	4	48	RTOG 0915 [112]
		Max Point Dose	40 Gy			
		<10 cc	30 Gy	5	40–60	RTOG 0813 [91]
		Max Point Dose	32 Gy			
Lung	Basic Lung Function	1500 cc	7 Gy	1	34	RTOG 0915 [112]
		1500 cc	11.6 Gy	4	48	RTOG 0915 [112]
		1500 cc	12.5 Gy	5	40–60	RTOG 0813 [91]
	Pneumonitis	1000 cc	7.4 Gy	1	34	RTOG 0915 [112]
		1000 cc	12.4 Gy	4	48	RTOG 0915 [112]
		1000 cc	13.5 Gy	5	40–60	RTOG 0813 [91]

Abbreviations: PTV, planning target volume; RTOG, Radiation Therapy Oncology Group.

#### 11.3.2. Target Delineation

All at-risk structures/organs should be contoured individually, with planning organ at risk volume (PRV) margins allowed for intrafraction movement, to avoid preventable complications [35,57,59,92,98]. When possible, care should be taken to avoid including immediately adjacent OARs in the target volume [114]. In general, parallel OARs require a determination of the critical organ volume and threshold dose required to avoid end-organ damage [57], whereas for serial OARs maximum point doses are of more clinical concern, and more protracted regimens may be required to avoid catastrophic complications [57].

#### 11.3.3. Plan Optimization/Beam Arrangement/Weighting

Dose distributions can be altered at the time of plan optimization to reduce doses to at-risk structures. Use of SBRT beam angle/weighting optimization with tight aperture margins is crucial for creating a sharp dose gradient that provides adequate target coverage while avoiding overdosing critical structures [97]. Planning techniques, such as using RapidArc treatment delivery, increasing the number of non-coplanar beam angles, and/or using alternative beam arrangements and beam weighting, have been shown to reduce dose to the chest wall toxicity [83,89,98,115]. Use of alternative dose constraints should also be considered, including the CI and R50% (defined as a ratio of the 50% prescription IDL to PTV volume), which have been recently adopted by the RTOG in their most recent protocols to facilitate plan optimization [116].

Caution must be exercised when using published dose constraints, as uncertainty exists regarding the effect of dose and fractionation on toxicity such that tolerances may not be readily extrapolated from the conventionally fractionated to the hypofractionated setting [23]. In addition, practitioners must be aware of the curative potential of early stage lesions treated with SBRT, as the risk of low grade toxicity may not warrant reduction in target SBRT doses [23]. With increasing application of SBRT in the central lung region, improved delineation of risk will be necessary to prevent avoidable complications [23].

Moreover, advances in plan optimization may also improve outcomes while minimizing toxicity. With a technique developed by Dong *et al.*, the highly non-coplanar 4π planning algorithm utilizes simultaneous optimization of PTVs and OARs, including specific dose constraints for lung V_20_, V_10_, and V_5_ to arrive at a final plan. Using this technique, improvements were seen compared to standard IMRT and VMAT plans, with improved target dose coverage and reduced doses to at OARs, allowing for potential dose escalation [117].

#### 11.3.4. Treatment Delivery

Lastly, new technologies in treatment delivery also hold the potential for reducing radiation toxicities. Real-time tumor tracking via 4D SBRT may allow better dose delivery to the tumor as it moves throughout respiration, thus reducing ITV and PTV margins, and consequently adjacent normal tissue dose [118]. Helical tomotherapy (HT) may improve OAR sparing for those in very close proximity to the PTV, as is the case with central lung tumors [119]. Additionally, flattening filter-free (FFF) linear accelerators, capable of delivering dose rates up to four times that of conventional linear accelerators, may allow for shorter treatment delivery times and thus reduce the opportunity for intrafraction motion [120] and exposure of normal tissue to scattered dose outside the field [121]. Volume-adapted dosing strategies hold promise for decreased radiotherapy intensity for smaller tumors with similar tumor control, and consequently, reduced dose to OARs [122].

## 12. Conclusions

SBRT has become a standard treatment option for early stage, node negative NSCLC, largely due to high local control rates and favorable toxicity profile compared with both surgical and non-surgical approaches. With excellent tumor control rates, the recent focus of lung SBRT strategies has been to limit toxicity while treating increasingly complex patients with a diverse array of presentations. Improvements in our understanding of patient-specific risk factors and prognosticators of outcomes will play a larger role in evaluating patients for SBRT candidacy, as well as stratifying those amenable to escalation and de-escalation of treatment. Traditional and novel dosimetric constraints will continue to be redefined, particularly in the re-irradiation setting, as improvements are made in our understanding of critical organ tolerances and dose-volume thresholds required to deliver safe treatments. Developments in radiation planning and delivery are also expected, which will complement developments in our understanding of the radiobiology of hypofractionated ablative radiotherapy on both tumor and normal tissue. Although we expect larger, prospective multi-institutional experiences to validate previous work, broaden our understanding, and present new challenges, at present it remains imperative to recognize current toxicity limitations of SBRT and treat patients accordingly.
